# 89976 ASSESSING PROTEIN BIOMARKERS ROLE IN CVD RISK PREDICTION IN PERSONS LIVING WITH HIV (PWH)

**DOI:** 10.1017/cts.2021.526

**Published:** 2021-03-30

**Authors:** Sandra Safo, Lillian Haine, Jason Baker, Cavan Reilly, Daniel Duprez, Jim Neaton, Jiuzhou Wang, Mamta K. Jain, Alejandro Arenas Pinto, Therese Staub, Mark Polizzotto

**Affiliations:** 1 University of Minnesota; 2 Hennepin Healthcare; 3 University of Minnesota School of Public Health, Biostatistics; 4 UT Southwestern Medical Center; 5 University College London; 6 Centre Hospitalier de Luxembourg; 7 The Kirby Institute, University of New South Wales Sydney

## Abstract

ABSTRACT IMPACT: Our findings could potentially identify CVD at-risk persons living with HIV who might benefit from aggressive risk-reduction. OBJECTIVES/GOALS: PWH have higher rates of CVD than the general population yet CVD risk prediction models rely on traditional risk factors and fail to capture the heterogeneity of CVD risk in PWH. Here we identify protein biomarkers that are able to discriminate between CVD cases and controls in PWH, and we assess their added benefit beyond traditional risk factors. METHODS/STUDY POPULATION: We analyzed 459 baseline protein expression levels from five OLINK panels in a matched CVD (MI, coronary revascularization, stroke, CVD death) case-control study with 390 PWH from INSIGHT trials (131 cases, 259 controls). We formed 200 datasets via bootstrap. For each bootstrap set, a two-component partial least squares discriminant model (PLSDA) was fit. The importance of each variable in the discrimination of cases and controls in the PLSDA projection was assessed by the variable importance in projection (VIP) score. Proteins with average VIP scores > 1 were used in penalized logistic regression models with elastic net penalty, and proteins were ranked based on the number of times the protein had a nonzero coefficient. Proteins in the top 25th percentile were considered to have high discrimination. RESULTS/ANTICIPATED RESULTS: Participants had mean age 47 years, 13% were females, 4.9% had CVD at baseline and 69% were on ART at baseline. Eight proteins including the hepatocyte growth factor and interleukin-6 were identified as able to distinguish between CVD cases and controls within PWH. A protein score (PS) of the top-ranked proteins was developed using the bootstrap (for weights) and the entire data. The PS was found to be predictive of CVD independent of established CVD and HIV factors (Odds ratio: 2.17 CI: 1.58-2.99). A model with the PS and traditional risk factors had a 5.9% improvement in AUC over the baseline model (AUC=0.731 vs 0.69), which is an increase in model predictive power of 18%. Individuals with a PS above the median score were 3.1 (CI: 1.83- 5.41) times more likely to develop CVD than those with a protein score below the median score. DISCUSSION/SIGNIFICANCE OF FINDINGS: A protein score developed improved discrimination of PWH with CVD and those without, and helped identify PWH with high risk for developing CVD. If validated, this score and/or the individual proteins could be used in addition with established factors to identify CVD at-risk individuals who might benefit from aggressive risk-reduction.

